# Prenatal Exposures to Multiple Thyroid Hormone Disruptors: Effects on Glucose and Lipid Metabolism

**DOI:** 10.1155/2016/8765049

**Published:** 2016-02-17

**Authors:** Deborah Molehin, Marloes Dekker Nitert, Kerry Richard

**Affiliations:** ^1^School of Medicine, The University of Queensland, Herston, QLD 4029, Australia; ^2^UQ Centre for Clinical Research, The University of Queensland, Herston, QLD 4029, Australia; ^3^Conjoint Endocrine Laboratory, Chemical Pathology, Pathology Queensland, Queensland Health, Herston, QLD 4029, Australia; ^4^School of Biomedical Sciences, Queensland University of Technology, Brisbane, QLD 4001, Australia

## Abstract

*Background.* Thyroid hormones (THs) are essential for normal human fetal development and play a major role in the regulation of glucose and lipid metabolism. Delivery of TH to target tissues is dependent on processes including TH synthesis, transport, and metabolism. Thyroid hormone endocrine disruptors (TH-EDCs) are chemical substances that interfere with these processes, potentially leading to adverse pregnancy outcomes.* Objectives.* This review focuses on the effects of prenatal exposures to combinations of TH-EDCs on fetal and neonatal glucose and lipid metabolism and also discusses the various mechanisms by which TH-EDCs interfere with other hormonal pathways.* Methods.* We conducted a comprehensive narrative review on the effects of TH-EDCs with particular emphasis on exposure during pregnancy.* Discussion.* TH imbalance has been linked to many metabolic processes and the effects of TH imbalance are particularly pronounced in early fetal development due to fetal dependence on maternal TH for proper growth and development. The pervasive presence of EDCs in the environment results in ubiquitous exposure to either single or mixtures of EDCs with deleterious effects on metabolism.* Conclusions.* Further evaluation of combined effects of TH-EDCs on fetal metabolic endpoints could improve advice provided to expectant mothers.

## 1. Introduction

Pregnancy results in many physiological changes that have significant effects on thyroid status [1]. The fetus relies exclusively on maternal thyroid hormone (TH) in early pregnancy for growth, neurodevelopment, and the regulation of metabolic processes [2, 3]. Dysregulation in glucose and lipid metabolism has been associated with many metabolic syndromes including gestational diabetes mellitus (GDM) and TH imbalance is a contributing factor to these diseases [4, 5]. The global prevalence of GDM and obesity is rising in the obstetric population and their effects on maternal and fetal outcomes are well documented [6–8].

Caloric intake and personal lifestyle are strongly associated with obesity and metabolic syndrome; however, there is a growing concern that a subgroup of endocrine disrupting chemicals (EDCs) known to specifically alter TH regulation (TH-EDCs) may contribute to the prevalence of metabolic syndromes by altering signalling pathways involved in glucose and lipid homeostasis during pregnancy. The risk of exposure to TH-EDCs is rising with exposure to multiple TH-EDCs more common than exposure to single chemicals. Several studies have reported the effects of exposure to individual EDCs and this has been reviewed elsewhere [9]. This review discusses the effects of combinations of TH endocrine disruptors on the regulation of glucose and lipid metabolism during pregnancy.

## 2. Method

For this narrative review conducted between 2014 and 2015, the PubMed (US National Library of Medicine, National Institutes of Health) and Google Scholar databases were interrogated with the following key words and phrases: TH endocrine disruptors and pregnancy/early or late pregnancy; prenatal effect of TH endocrine disruptors on glucose/lipid metabolism; mechanism of action of TH endocrine disruptors; placental transfer/biotransformation of TH endocrine disruptors. All study types including randomized controlled trials, case-control, human, and animal studies were considered and results are restricted to English only. The articles were grouped according to the effects of the TH-EDCs on related endpoints.

## 3. Results and Discussion

### 3.1. Thyroid Hormone Synthesis, Secretion, and Metabolism

**Figure 1:**
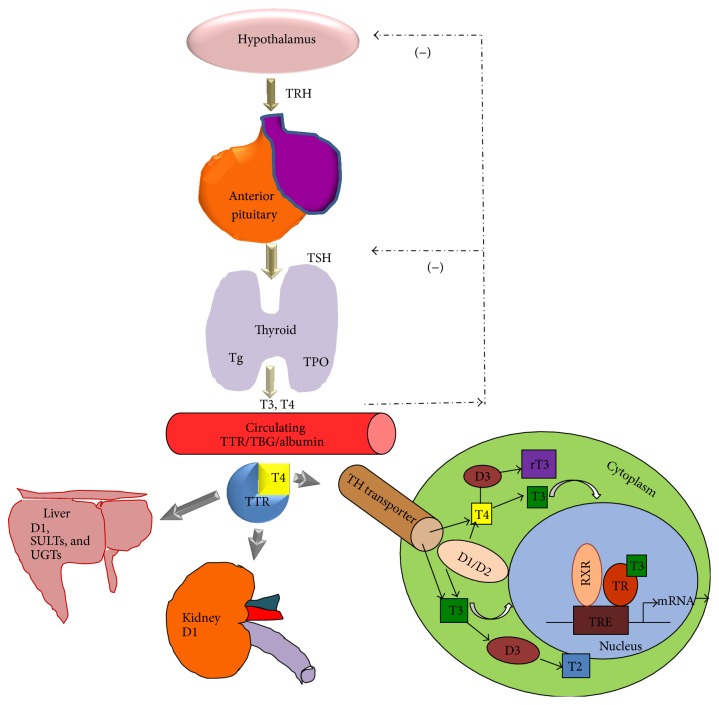
Thyroid hormone synthesis and secretion regulated through a negative feedback loop. In the hypothalamus, Thyrotropin- (TSH-) releasing hormone (TRH) stimulates the anterior pituitary gland to secrete thyroid stimulating hormone (TSH) which then initiates thyroid hormone (TH) synthesis and release from the thyroid gland by the action of thyroid peroxidase enzyme (TPO) on thyroglobulin (Tg). TRH and TSH are inhibited by negative (−) feedback of the thyroid hormones. Thyroxine (T4) and triiodothyronine (T3) are released into the circulation where they bind thyroid hormone binding proteins, namely, transthyretin (TTR), thyroxine binding globulin (TBG), and albumin. These complexes are then transported into cells via TH transporters. In the cell, Types 1 and 2 deiodinase enzymes convert T4 to T3, which then enters the nucleus and binds with thyroid hormone receptors (TRs) which in turn bind other nuclear receptors (e.g., retinoid X receptor (RXR)). These receptor complexes then bind thyroid hormone responsive elements (TREs) on target genes which results in the transcription of the DNA sequence to messenger ribonucleic acid (mRNA). Deiodinase Type 3 (D3) also regulates thyroid hormones by converting T4 and T3 to reverse T3 (rT3) and 3,5-diiodo-L-thyronine (T2), respectively. In the liver, deiodinase Type 1 (D1) enzyme is involved in both T3 production and clearance of plasma rT3. Thyroid hormone is also metabolized by conjugation to sulphate (by sulfotransferases (SULTs)) and glucuronic acid (by UDP-glucuronosyltransferase (UGTs)). Conjugation increases the water solubility of TH, facilitating its rapid degradation. In the kidney, Type 1 (D1) deiodinase is also involved in T3 production and excretion of thyroid hormones.

The THs thyroxine (T4) and triiodothyronine (T3) are synthesized and secreted by the thyroid gland. Thyroid epithelial cells synthesize thyroglobulin, which provides tyrosine residues that are iodinated to iodotyrosine by thyroid peroxidase (TPO) to form T4 and T3 (20% of total T3 is made by the thyroid gland) [10]. Maintenance of blood TH levels occurs through a hypothalamic-pituitary-thyroid axis feedback mechanism [10]. Basically, TSH-releasing hormone (TRH) in the hypothalamus stimulates thyroid stimulating hormone (TSH) secretion from the anterior pituitary, which in turn initiates TH synthesis and release from the thyroid gland. TH also acts at the transcriptional level to suppress the synthesis of TRH and TSH (Figure 1).

**Figure 2:**
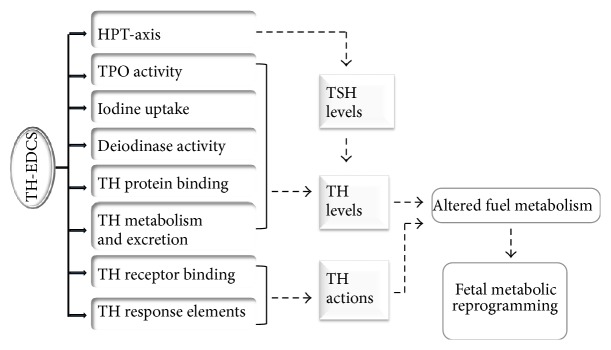
TH-EDCS targets and effect on fetal fuel metabolism. Thyroid hormone endocrine disrupting chemicals (TH-EDCs) disrupt the thyroid economy by altering the hypothalamus, anterior pituitary, and thyroid axis (HPT-axis) which results in altered thyroid stimulating hormone (TSH) levels and in turn the thyroid hormone (TH) levels. TH-EDCs also interfere with the synthesis of TH by inhibiting thyroid peroxidase (TPO) activity, iodine uptake, and deiodinase activity, TH binding to transport proteins, and TH metabolism and excretion which all result in the alteration of TH levels. TH-EDCs inhibit TH binding at the TH receptors and at the TH response elements (TREs); this results in the inhibition of TH action on target genes. Alterations in the TH levels as well as its action lead to altered fuel metabolism and eventually in fetal metabolic reprogramming.

THs are highly lipophilic and are secreted into the blood stream where they are bound by the TH distribution proteins thyroxine binding globulin (TBG), transthyretin (TTR), or albumin [11]. TH cellular uptake is regulated by cell specific expression of TH transporters including organic anion-transporting polypeptides (OATPs), large neutral amino acid transporters (LATs), and monocarboxylate transporters 8 and 10 (MCT8, MCT10) (Figure 2).

Once inside the cell, the deiodinase enzymes (Type 1 (D1), Type 2 (D2), and Type 3 (D3)) regulate the conversion of T4 to either active T3 (D1 and D2) or inactive reverse T3 (rT3) (D1 and D3) by the removal of a specific iodine atom [12]. Metabolizing enzymes such as uridine 5′-diphospho-glucuronosyltransferases (UDP-GT) and sulfotransferases also regulate TH bioavailability by rendering THs more water soluble and more easily excreted [13].

THs converted to T3 in the cell can either activate or repress gene transcription by binding to nuclear TH receptors (TRs) which are DNA-binding transcription factors that bind specific thyroid hormone response elements (TREs) in the regulatory region of target genes [14]. TRs may bind TREs as homodimers but also commonly bind as heterodimers with other nuclear receptors such as the retinoid X receptor (RXR) [15].

A person is described as euthyroid when the thyroid gland is functioning normally resulting in normal steady state levels of serum THs and TSH. Hypothyroidism results when the thyroid gland does not produce adequate amounts of TH resulting in low serum TH levels and elevated TSH. Hyperthyroidism results from the thyroid gland producing too much TH resulting in high TH and suppressed TSH in the serum. Both hypo- and hyperthyroidism commonly result from autoimmune disease attack of TPO (hypo- and hyper-), thyroglobulin (hypo-), TSH receptor antibodies, and thyroid stimulating immunoglobulin (hyper-). When serum TH levels fall within the normal range but TSH levels are not normal (autoantibodies are also often present), this signifies the early stages of thyroid disease and is described as a subclinical state [38]. TH disrupting chemicals would clearly have different effects on individuals with different types of thyroid disorder.

### 3.2. Thyroid Hormone Metabolism and Pregnancy

TH levels are markedly altered by the hormonal and metabolic demands of normal pregnancy [39]. The placenta secretes a glycoprotein hormone called human chorionic gonadotropin (hCG), which possesses structural similarities to TSH and acts as a weak TSH receptor agonist [40]. Due to elevated levels of estrogen and hCG in pregnancy, a marginal increase in the basal TSH and serum TBG as well as alterations in TH levels and peripheral metabolism of maternal TH is believed to alter the thyroid hormone system [41].

The maternal thyroid gland accommodates the demands of pregnancy and a growing fetus by increasing hormonal output. The human fetal thyroid can secrete TH from 16 weeks of gestation [42], whereas prior to this the fetus is dependent on transplacental supply of maternal T4. In the first trimester, there is a parallel increase in total T4 concentration in the maternal and fetal compartments. Lower T3 but higher rT3 concentrations were observed in coelomic and amniotic fluids [43]. This was attributed to high fetal D3 and sulfotransferase activities and confirmed by reports of high D3 activities in fetal hepatic and placental tissues [44, 45]. In addition, due to lower levels of binding proteins such as TTR in the human fetus, a higher proportion of maternally transferred T4 exists as free T4 in the coelomic fluid cavities [43]. TTR is produced and secreted by the human placenta [46] and binds T4 with high affinity and may transport maternal T4 to the fetus.

#### 3.2.1. Effects of Thyroid Hormones on Glucose Metabolism

Glucose metabolism is altered during pregnancy to guarantee adequate delivery of nutrients to the growing placenta and fetus. In pregnancy, maternofetal glucose transport is largely dependent on the expression of one or more member of the facilitative family of glucose transporters (GLUTs) in the placenta. Increased fat mass in overweight and obesity is associated with prepregnancy insulin resistance, which is compounded by insulin resistance of pregnancy [47, 48]. In late pregnancy, insulin resistance is associated with hepatic gluconeogenesis and decreased utilization of glucose by peripheral tissues which contribute to meeting the increasing placental and fetal energy demands [49, 50]. With higher prepregnancy insulin resistance, for example, in obesity, the additional pregnancy-induced insulin resistance cannot always be compensated for by increased insulin secretion resulting in overt hyperglycemia. Gestational diabetes mellitus (GDM) is hyperglycemia first detected during pregnancy and may affect up to 17.8% of pregnancies [51]. The etiology of GDM has been attributed to genetic predisposition, insulin resistance, and altered beta cell function [52–54].

TH affects many aspects of glucose metabolism: circulating insulin levels and counter-regulatory hormones; hepatic gluconeogenesis and glycogenolysis; and intestinal glucose uptake. TH regulates the transcription of several genes involved in glucose metabolism. At the tissue level, TH actions on glucose metabolism are regulated by transmembrane transporters, ligand-dependent TH receptors, and deiodinases [55].

Deranged TH levels in pregnancy are associated with adverse maternal and fetal metabolic outcomes [4, 56, 57]. Excess TH increases basal metabolic rate with a concomitant increase in the demand for glucose [58]. In response to excess TH, pancreatic *β* cells continuously secrete insulin even in the absence of stimulatory glucose concentrations. This results in hyperinsulinemia and subsequently insulin resistance [59]. Untreated hyperthyroidism, although uncommon in pregnant women, may lead to restricted fetal growth, stillbirth, or preterm delivery due to reduced availability of nutrients to the fetus [60, 61]. Furthermore, in healthy pregnant women with normal prepregnancy thyroid function, lower free T4 concentrations and a higher conversion of free T4 to free T3 are positively correlated with elevated glucose levels after oral glucose load as well as higher fasting insulin when adjusted for BMI [56]. This suggests that variations in TH levels within euthyroid concentrations also impact glucose metabolism in pregnancy.

In contrast, evidence of decreased glucose production and utilization has been observed in overt and subclinical hypothyroidism [62]. TH replacement therapy in patients with subclinical hypothyroidism resulted in a partial reversal to the euthyroid state with significant reductions in glucose-stimulated-insulin secretion (GSIS) [62]. This implies that poorly managed hypothyroidism could result in high nutrient availability for the fetus, leading to large gestational age infants predisposed to obesity later in life. Animal studies of maternal hypothyroidism have reported impaired brain development perhaps due to altered expression of GLUT protein isoforms in the placenta and fetal brain [63].

#### 3.2.2. Effects of Thyroid Hormones on Lipid Metabolism

Maternal serum lipid concentrations increase as pregnancy progresses [64]. The dynamics of lipid metabolism during pregnancy vary with gestational age: accumulation of fat in early pregnancy (anabolic phase), shift to increased insulin resistance, decreased adipose tissue lipoprotein lipase (LPL) activity, and increased lipolysis in late pregnancy (catabolic phase) [65]. The rise in maternal insulin resistance allows more glucose to be transported to the fetus in late pregnancy while the mother utilizes lipids as her main energy source. The placental transport of fatty acids to the growing fetus is mediated by lipoprotein receptors, lipid carrier proteins, and actions of lipase enzymes [65]. The exact mechanisms of placental lipid transport are however still unclear.

Maternal lipid metabolism in normal and complicated pregnancies has recently been reviewed [66]. In women with GDM, the higher levels of insulin resistance are associated with increased adipose tissue lipolysis and higher free fatty acids and triglycerides in maternal serum, increasing fetal nutrient availability resulting in fetal overgrowth [67].

TH is a main regulator of lipid metabolism. The expression of TH receptor isoforms is an important determinant of its actions on lipid metabolism [68]. The overall effects of TH on lipid metabolism are the sum of its actions primarily in the liver and adipose tissue. In the liver, TH stimulates lipogenesis, whereas in adipose tissue TH stimulates lipolysis [68]. TH increases hepatic cholesterol uptake and synthesis mainly not only by inducing the transcription of the LDL-receptor [69] but also by reducing apolipoproteins B48 and B100 [70], which is associated with increased hepatic triglyceride production. The higher TH levels in pregnancy may contribute to the increased lipid levels observed in the maternal circulation. Effects of TH and its receptors on rodent lipid metabolism have been reviewed [71].

Thyroid dysfunction has been associated with abnormal lipid profiles. During normal pregnancy, altered TH levels have been associated with a less favorable metabolic phenotype in pregnant women with normal thyroid function prepregnancy [56, 72]. Hyperthyroidism is characterised by an increased turnover of LDL cholesterol resulting in decreased plasma lipid levels [73]. In contrast, hypothyroidism in late pregnancy has been associated with enhanced cholesterol levels and decreased triglycerides and HDL-cholesterol levels in rats [74]. Because of the importance of TH in regulating glucose and lipid metabolism in pregnancy and thereby the growth of the baby, substances that affect TH may have large effects on the health and development of mother and infant (Table 1).

### 3.3. Thyroid Hormone Endocrine Disrupting Chemicals

Exogenous substances capable of interfering with the structure or function of the endocrine system are known as endocrine disrupting chemicals (EDCs). As with other hormones, TH is a target of EDCs and many studies have identified chemicals that alter TH homeostasis. TH-EDCs may be clustered into two main groups based on their biodegradability and bioaccumulation in the environment. Nonpersistent organic chemicals (N-POCs) are widespread in the environment but are nonlipophilic and do not bioaccumulate. Persistent organic chemicals (POCs) are highly stable lipophilic compounds that bind to adipose tissue in living organisms and bioaccumulate up the food chain. N-POCs and some POCs are rapidly metabolized by enzymes and eliminated from the body. Organochlorine (OC) and organophosphate (OP) pesticides are highly lipophilic; OPs are unstable and therefore more readily metabolized than OCs which have been found to accumulate in adipose tissue [75].

TH-EDCs are ubiquitous and originate from chemical additives used as flame retardants; synthetic plasticizers and solvents used in food packaging, polyvinyl chloride tubing, medical equipments, pesticides, toys, personal products, adhesives, powder paints, and dental sealants; antimicrobial compounds used in household detergents; toxic by-products of combustion processes; insulating materials for electrical equipment such as transformers and capacitors, heat transfer systems, hydraulic fluids, and lubricants; airbag inflation systems, fireworks, nitrate fertilizers, matches, and oxidants in propelling rockets and missiles; and synthetic and naturally predominant compounds in soy rich foods [9, 16, 76]. Humans come in contact with these chemicals through ingestion, inhalation, dermal exposure to contaminated substances, and intravenous and parenteral absorption from medical devices containing phthalates.

#### 3.3.1. Mechanisms of Action

TH-EDCs interfere with TH and TSH signalling through many pathways in many different species: altering deiodinase activity [25, 28, 77], inhibiting TH excretion or metabolism [26, 29], blocking iodine uptake by thyroid cells [33], competitively binding the thyroid transport protein TTR, the inhibition of human TPO [36, 37], and acting as an antagonist of complexes from the thyroid hormone responsive elements (TREs) [19, 78].

Certain TH-EDCs such as brominated flame retardants, hydroxylated polychlorinated biphenyls (PCBs) metabolites, and dioxin (PCDD) share structural similarities with TH and bind with the high affinity TH transport protein TTR [79], consequently inhibiting T4-TTR binding. In serum samples from polar bears, T4 binding sites on TTR displayed a higher affinity for halogenic phenols and PCBs. Supraphysiological levels of T4 were unable to displace these compounds when bound to TTR [80]. In pregnant rats, reduced fetal plasma and brain total T4 levels in response to prenatal exposure to hydroxylated PCBs were hypothesized to be due to the binding to TTR [77]. Many TH-EDCs not only affect TH but also interfere with the actions of other hormones acting through nuclear receptors such as sex hormones (estrogen, progesterone, and androgen) or by interacting with their respective nuclear receptors (ER, PR, and AR).* In vitro* toxicology studies of non-dioxin-like PCBs on humans revealed that PCB related compounds (congener) 168 and 125 completely inhibited T4-TTR binding because of their structural similarity to T4, but they also had very high androgen-inhibitory potencies. PCB-168 exhibited weak antiestrogenic activities whereas PCB-125 exhibited ER-mediated activity. On the other hand, PCB-104 had high AR-antagonistic potencies and was the most effective congener with estrogenic properties of all congeners studied [78]. Exposures to TH-EDCs in pregnancy have been associated with alterations in TH regulation and adverse pregnancy outcomes (Table 2).

The effects of TH-EDCs on the levels of TH or its receptor will affect downstream signalling including metabolic pathways. Bisphenol A functions as a selective TR-beta antagonist* in vivo* [81]. TR-beta is the main TR isoform in both liver and adipose tissue and BPA exposure could therefore reduce both lipogenesis and lipolysis reducing lipid availability in the circulation. Furthermore, the interactions between the TR and other nuclear receptors such as the RXR and FXR receptors further complicate the effects of EDCs since many TH-EDCs such as BPA independently affect other nuclear receptors which also affect lipid and glucose metabolism [82]. It may be possible to predict the effects of combinations of EDCs based on their individual TH signalling targets. A summary of TH signalling targets of different TH-EDCs is presented in Table 3.

#### 3.3.2. Significance of the Effects of Multiple Endocrine Disruptor Exposures over Single Exposures

In modern society, most individuals will have been exposed to mixtures of EDCs rather than single EDC. Therefore, the study of the effects of combined chemicals is critical to reach meaningful conclusions on the plausible role of these chemicals on human health. Effects of EDCs can either be additive, synergistic, or antagonistic since chemicals may interact with one another to modify the nature of the toxic effect. For instance, human studies [83] revealed significant associations between increased levels of PCBs, dichlorodiphenyldichloroethylene (DDE), and hexachlorobenzene (HCB) with adverse thyroid volumes and multiple metabolic disorders, especially in older subjects. Complex mixtures may therefore result in more or additional deleterious effects on metabolism (Figure 2).

### 3.4. Prenatal Exposures to TH-EDCs

Prenatal exposure to EDCs continues to pose serious health risks to developing fetus and children as evidence of adverse effect on birth outcomes, childhood obesity, and intellectual disability are increasing [4, 84, 85]. More importantly, because organogenesis begins at the time when the fetus is solely dependent on maternal TH supply, early life exposure to TH-EDCs may lead to adverse short or long term health outcomes due to fetal reprogramming [86, 87].

#### 3.4.1. Placental Transfer and Biotransformation of TH-EDCs

**Figure 3:**
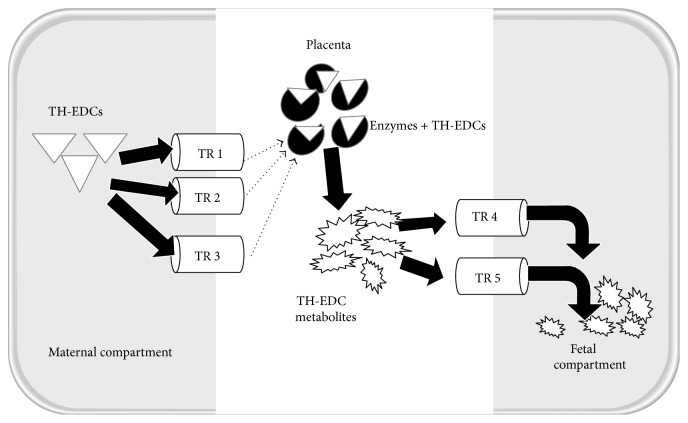
Placental transfer and biotransformation of TH-EDCs. Thyroid hormone endocrine disrupting chemicals (TH-EDCs) enter the placenta from the maternal compartment via various transporters located at the maternal interface of the placenta and are biotransformed by metabolizing enzymes to various metabolites that then enter the fetal compartment through transporters located at the fetal interface of the placenta. TR indicates transporters.

The placental barrier is not impervious to TH-EDCs as many of them have been measured in human fetal cord blood [88], neonatal meconium [89], rat fetal serum [22], and human amniotic fluid [17]. TH-EDCs are able to traverse the placental barrier by diffusion or via an active transporter (such as OATPs) either as pure or as biologically transformed chemicals (through conjugation of chemicals by placental metabolizing enzymes such as sulfotransferases (SULTs) and UDP-glucuronosyl transferases (UGTs)) (Figure 3). Biotransformation of chemicals by metabolizing enzymes makes the chemicals fit for excretion and may result in inactivation or increased toxicity. The biotransformed compounds can be extruded into the maternal circulation for excretion via placental transporters such as multidrug resistant-associated proteins. Expression of metabolizing enzymes and transporters has been shown to vary with gestational age in human [90] and animal placentas [91, 92]. The majority of the enzymes and transporters are expressed throughout human pregnancy with decreasing expression observed as pregnancy advances (e.g., UGT1 [93, 94]: P-glycoprotein (P-gp) [90]) whereas others are expressed during late pregnancy and continued during postnatal period [95]. The decline in the expression of transporters with gestational age is further corroborated by the increased rate of placental transfer and amount of P-gp substrates during late pregnancy compared to early pregnancy [96]. Some TH-EDC chemicals may alter other metabolic pathways thereby modulating the effect of metabolizing enzymes on endogenous substrates like steroid hormones [92] or other xenobiotics [97].

*Ex vivo* studies on human term placentas demonstrated that environmentally relevant levels of BPA in mothers freely diffuse across the placenta in an unconjugated form suggesting that SULTs and UGTs play a minor role in the transplacental transport of BPA. This was attributed to the low expression of placental enzymes involved in metabolizing the chemicals towards the end of pregnancy [98]. A similar study on term human placentas revealed that genistein, a naturally occurring phytoestrogen was able to traverse the placental barrier although only a small proportion was biotransformed by metabolizing enzymes in the placenta [99]. BPA and genistein both share similar metabolic pathways as well as estrogen disrupting effects [97]. UGT activity has been shown to be higher in human first trimester placenta compared to term placentas [94] and to decrease as pregnancy advances in humans [93]. This implies that the fetus is more protected from the toxic effect of TH-EDCs by UGT enzymes in early pregnancy than in late pregnancy, which might be a consequence of the vulnerable detoxification system in the infant in early pregnancy. This is different from animal models; here BPA actively traversed the rat term placenta predominantly in its conjugated form and was deconjugated by *β*-glucuronidase enzymes in the rat fetus [100]. In addition to the low UGT activity observed in the rat fetus, high levels of Oatp4a1 and Mrp1 transporters were expressed at the maternal and fetal interfaces of the placenta, respectively, suggesting that conjugated BPA is transferred from mothers by Oatp4a1 to the growing fetus by Mrp1 [100] leading to toxic effects of chemicals on the fetus.

#### 3.4.2. Effects on Adipogenesis

Prenatal exposures to mixtures of TH-EDCs have revealed diverse ways by which chemicals alter TH homeostasis. Perinatal exposures to low doses of BPA increase abdominal adipocyte tissue mass and correlate with hyperlipidemia in a dose-response manner in mice [101]. However, exposures to low concentration of BPA had no adipogenic effect on murine mesenchymal stem cells* in vitro* [102]. Although the nonmonotonic effect of BPA, characterized by high responses at low and high exposure levels, is well known, it is worth mentioning that a much lower BPA concentration was administered to the pregnant mice than the mesenchymal stem cells. This discrepancy may be a result of the insulin resistant state in pregnancy, which is related to decreased adipogenesis. In normal pregnancy, placenta-derived hormones induce a state of insulin resistance with the aim of maintaining adequate energy supplies to the placenta and developing fetus. Insulin is known to promote lipogenesis in adipose tissue while suppressing lipolysis therefore an insulin resistant state will result in reduced lipogenesis while at the same time favoring lipolysis. A study of individual chemical effects on adipogenesis in animal models demonstrated an association between high doses of BPA and decreased adipocyte and lipid levels whereas diethylhexylphthalate (DEHP) and tributyltin (TBT) were linked to enhanced adipogenesis [102]. However, when studied together, the negative effect of BPA on adipogenesis was outweighed by the positive effect of DEHP and TBT on the proliferation of adipocytes at high concentrations with increased adipogenesis even though the effect was not as profound as observed with individual DEHP and TBT chemicals. No adipogenic effect was observed at low concentrations of the chemical mixture [103]. This suggests that the effect of chemical mixtures cannot be predicted from outcomes from individual chemicals due to varied mechanisms of action. In this case, BPA signals through the regulation of estrogen even though studies of BPA itself have shown that BPA can alter TH regulation [16]. On the other hand, DEHP and TBT both activate the peroxisome proliferator-activated receptors gamma (PPAR*γ*) signalling pathway.

#### 3.4.3. Effects on Preterm Birth

Altered TH levels and postnatal thyroid function have been reported in preterm infants [104]. Epidemiological studies have revealed inverse or positive associations between maternal urinary BPA and mono-2-ethyl-5-hydroxyhexyl phthalate (MEHHP) with TH levels in adults and adolescents, respectively [105, 106]. Also increases in BPA and MEHHP are associated with significant reductions in gestation in male offspring specific pregnancies in one [107] but not in another study [108]. These differences may be due to the small sampling population used in the study.

#### 3.4.4. Effects on Birth Weight

Higher maternal prepregnancy BMI and higher gestational weight gain are correlated with higher birth weight and fat mass at birth and increased BMI in young and adult offspring [109, 110]. In addition, maternal preexisting diabetes and gestational diabetes have been associated with increased birth weight and development of later childhood obesity [111]. In a monotonic relationship, an increase in the dose of a chemical is attended by a corresponding increase in the effect of the chemical on the observed endpoint. Likewise, there is a corresponding decline in the effect with decreasing the doses of the chemicals. In male neonates, monotonic relationships were observed between phenols and birth weights; however, a U-shaped nonmonotonic association existed between phthalate metabolites and birth weights [112].

#### 3.4.5. Long Term Effects on Obesity

Fetal exposures to phthalates and dichlorodiphenyldichloroethylene (DDE) have been associated with increased BMI and head circumference (HC) during the first year after birth in a nonmonotonic manner [88]. Animal studies revealed that exposures to a low dose of MEHP during pregnancy significantly increased body weight and fat pads of male offspring at 60 days after delivery, as well as serum cholesterol, triacylglycerol (TAG), and glucose levels in mice [113]. This demonstrates that low-dose effects cannot be predicted from outcomes at higher doses [114]. The specific effect on male offspring may be due to the interference of MEHP with male hormones as human epidemiological studies have revealed an association between MEHP and decreased steroid hormones in adult males [115]. Sex hormones have been linked to obesity [116] with positive relationship observed between androgen levels and BMI in females while a negative relationship exists between levels of androgen and small waist circumference in males [117]. In breast cancer cells, estrogen inhibits PPAR*γ* [118], but MEHP is an agonist of PPAR*γ* [119]. Hence, it can be speculated that, at low MEHP levels, the ratio of androgen to estrogen is high, therefore resulting in the observed obesity phenotype in male offspring. However, in female fetuses with higher estrogen to androgen levels, low level MEHP exposure would lead to estrogen inhibition of PPAR*γ* activity and lipid metabolism.

Perinatal exposure to polychlorinated biphenyls (PCBs) and dioxins and their effects on puberty have been reviewed [120]. Exposure to PCB/dioxin has been linked to increased obesity, disruption of sex hormone signalling, retardation in the growth, and development of sex organs in males and breasts in girls. PCB/dioxin exert their role on puberty by interacting with the AhR which interferes with hormonal systems [120]. Prenatal exposure to a combination of PCBs and dichlorodiphenyldichloroethylene (DDE) may predispose female offspring to obesity in overweight mothers [121]. Exposure to DDE alone in infants of normal-weight women leads to more weight gain in the first 6 months postnatally and high BMI in early childhood [122]. Polychlorinated dibenzo-p-dioxins (PCDDs) and polychlorinated dibenzofurans (PCDFs) were found to significantly affect birth weight among male infants but not among female infants [123]. However, dietary dioxin and PCB intake was not associated with the risk for small-for-gestational age neonates [124]. This implies that some chemicals may have differential gender effects by interacting with some gender specific hormone receptors and signalling pathways such as estrogen and androgens.

#### 3.4.6. Confounding Factors

Many studies have associated exposures of persistent organic pollutants with TH dysregulation [27, 28, 30]. The effects of some chemicals may be impacted by confounding factors such as maternal demographic and perinatal factors. Maternal gestational weight gain (GWG) has been implicated to influence the overall burden of persistent organic pollutants (POPs) in neonates [125]. In the anabolic phase of pregnancy, mothers use less of their stored fats, which would reduce the proportion of POPs that get liberated from fat stores. On the contrary, late pregnancy is marked by increased lipolysis, which may contribute to the increased release of compounds trapped in stored lipids, leading to an even higher exposure in the fetus. It could thus be that the level of exposure to POPs varies over the course of pregnancy.

A negative association was recorded between PBDE congeners and birth weight; however, when adjusted for maternal weight gain, these findings were no longer statistically significant. Also, no association was observed between PBDEs and birth length, head circumference, or gestational duration [126]. Exposure to PCBs and POPs during pregnancy has been linked to adverse effects on fetal growth [127–129], increased birth weight [130], and shortened length of gestation in humans [131]. Prenatal exposures demonstrated a link between DDE and HCB with early postnatal growth but not with PCB [132]. It is crucial that confounders be identified in studies to rule out false positive results and to determine their role in maternal and fetal outcomes.

## 4. Conclusions

The role of TH-EDCs on metabolic risk factors such as insulin resistance, glucose tolerance, and triglycerides and cholesterol levels has only recently become the subject of research. Early studies suggest that TH-EDCs may act via a variety of mechanisms and that studies using high doses of these chemicals are probably not good predictors of effects at low doses. Additionally, low doses of combinations of chemicals, which would be a better reflection of the current situation, have shown conflicting results on the endpoints investigated. Variations in the effects of TH-EDCs on fetal growth have been reported with respect to gender and gestational age. In order to provide better prenatal care and improved health outcomes, it is important that studies be carried out to evaluate the effects of TH-EDCs on lipid levels, insulin sensitivity, and glucose tolerance as they are significant underlying factors in the development of metabolic syndrome.

## Figures and Tables

**Table 1:** Hallmarks and effects of hyper-/hypothyroidism on fetal growth.

	Hyperthyroidism	Hypothyroidism
Metabolic rate	++	−
Glucose demand by tissues	++	−
Glucose stimulated insulin secretion	−	−
Hyperinsulinemia	++	N/A
Insulin resistance	++	N/A
Glucose disposal	−	−
Hepatic gluconeogenesis	++	++
Lipogenesis	−	++
Lipolysis	++	−
Fetal growth	−	++

++ symbolizes increase and − symbolizes a decrease, whereas N/A means not applicable.

**Table 2:** Risk assessment of TH-EDCs and their effect on TH regulation in pregnancy.

Endocrine disruptors	Thyroid hormone-EDCs	Sources of exposure	Mode of exposure	Target tissues	Effect on TH regulation	References
Nonpersistent organic chemicals	Phthalates	Medical equipment, pesticides, and cosmetics	Ingestion, inhalation, and dermal exposures	Placenta, cord blood, and neonatal meconium	Impaired iodine uptake, inhibition of TH homeostasis	[16–18]
	Bisphenol A	Food can linings, dental sealants, and plastics	Ingestion, dermal exposures	Serum, amniotic fluid, and placenta	Binds TTR and inhibits TPO, T3 antagonist	[16, 19–21]
	Triclosan	Clothing, cosmetics, and detergents	Ingestion, dermal exposures	Urine, serum, and breast milk	Alters TH actions	[22–24]

Persistent organic chemicals	Flame retardants	Furniture, electronics, house dust, and foods	Inhalation, ingestion	Serum, milk, and cord blood	Disrupt TH signalling, alter TH levels, and inhibit TH sulfotransferase, TSH, and deiodinase activities	[25–27]
	Dioxins	By-products of industrial and environmental processes	Ingestion of contaminated dairy products	Fat tissue, breast milk	Alter TH and TSH levels and TPO and bind TTR	[28, 29]
	Polychlorinated biphenyls	Insulating materials, heat-transfer systems, lubricants, and paints	Dermal exposures, ingestion, and inhalation	Placenta, serum, adipose, and breast milk	Alter TH and TSH levels, bind TTR, and alter TH-responsive genes	[30–32]
	Perchlorates	Inflation systems, fireworks, nitrate fertilizers, and oxidants in propelling rockets and missiles	Drinking contaminated water, ingestion, and inhalation	Breast milk, urine	Inhibit iodine uptake via sodium iodide symporter (NIS) and alter TH and TSH levels	[33–35]
	Phytoestrogens	Soy rich foods	Ingestion		Decrease iodine accumulation, inhibit TPO activity, act as TR agonist or antagonist, and alter TH levels	[36, 37]

**Table 3:** TH signaling targets of different classes of TH-EDCs.

TH-EDCs	Iodine uptake	Inhibition of TPO	Binding to TTR	TH levels	TSH levels	Deiodinase activity	Sulfotransferase activity	Transporter/receptor
PBDE				x	x	x	x	
PCB			x	x	x			x
TCDD							x	
Triclosan							x	x
Perchlorate	x			x	x			
Flavonoids	x	x		x				x
BPA		x	x	x			x	
Dioxin		x	x	x				x
Phthalates	x			x				
